# Treatment of *Helicobacter pylori* infected mice with *Bryophyllum pinnatum,* a medicinal plant with antioxidant and antimicrobial properties, reduces bacterial load

**DOI:** 10.1080/13880209.2016.1266668

**Published:** 2016-12-09

**Authors:** Laure Brigitte Kouitcheu Mabeku, Bertrand Eyoum Bille, Thibau Flaurant Tchouangueu, Eveline Nguepi, Hubert Leundji

**Affiliations:** a Microbiology and Pharmacology Laboratory, Department of Biochemistry, Faculty of Science, University of Dschang, Dschang, Cameroon;; b Gastroenterology Department, Laquintinie Hospital of Douala, Douala, Cameroon

**Keywords:** Gastroduodenal infection, anti-*Helicobacter pylori*, herbal drug

## Abstract

**Context:** Bryophyllum pinnatum (Lam.) Kurz (Crassulaceae) is a plant known for its antiulcer properties.

**Objective:** This study evaluates the anti-*Helicobacter pylori* activity of *Bryophyllum pinnutum* methanol extract with a mouse model and its antioxidant properties.

**Materials and methods:** Dried leaves of *Bryophyllum pinnutum* were extracted with methanol and ethyl acetate. Broth microdilution method was used to evaluate the anti-*Helicobacter* activity of extract samples *in vitro*. Swiss mice were inoculated with a suspension of *Helicobacter pylori* and divided into control group and four others that received 125, 250, 500 mg/kg of methanol extract or ciprofloxacin (500 mg/kg), respectively, for 7 days. *Helicobacter pylori* colonization and bacterial load of mouse stomach was assessed on day 1 and 7 post-treatment. The antioxidant activity of *Bryophyllum pinnutum* was evaluated through DPPH radical, hydroxyl radical and reducing power assay.

**Results:** Methanol extract showed a significant anti-*Helicobacter* activity with MIC and MBC values of 32 and 256 μg/mL, respectively. *Bryophyllum pinnatum* and ciprofloxacin reduced *H. pylori* colonization of gastric tissue from 100% to 17%. *Bryophyllum pinnatum* extract (85.91 ± 52.91 CFU) and standard (25.74 ± 16.15 CFU) also reduced significantly (*p* < 0.05) bacterial load of gastric mucosa as compared to untreated infected mice (11883 ± 1831 CFU). DPPH radical, hydroxyl radical and reducing power assays showed IC_50_ values of 25.31 ± 0.34, 55.94 ± 0.68 and 11.18 ± 0.74 μg/mL, respectively.

**Discussion and conclusion:** The data suggest that the methanol extract of *Bryophyllum pinnatum* could inhibit *Helicobacter pylori* growth, and may also acts as an antioxidant to protect gastric mucosa against reactive oxygen species.

## Introduction

*Helicobacter pylori* is the principal cause of type B gastritis, peptic ulcer disease, gastric adenocarcinoma and MALT-lymphoma, and has been classified as a Class I carcinogen by the World Health Organization (WHO) (Manyi-Loh et al. 2010). It is a virulent pathogen as evidenced by its low infective dose and high prevalence in human populations. The prevalence of *H. pylori* infection varies from 20 to 50% in industrialized countries to over 80% in developing countries (Ndip et al. 2004). Currently, the treatment of symptomatic individuals is done with a regimen containing two antimicrobial agents most commonly amoxicillin, tetracycline, clarithromycin or metronidazole along with a proton pump inhibitor or bismuth compounds (Manyi-Loh et al. 2010). However, *H. pylori* is still a difficult infection to eradicate as failure rate remains at 10–40% (Lai et al. 2006). A major factor to this failure is the growing resistance of the microbe to conventional antimicrobial agents (Tanih et al. 2010). An alarming 100% resistance has been reported against metronidazole, one of the drugs used in the treatment regimen in some countries of the developing world (Aboderin et al. 2007). Other factors include high cost of combination therapy and availability, treatment side effects, poor patient compliance, and antibiotic inactivation by pH (Romano & Cuomo 2004). Thus developing new anti-*Helicobacter* compounds is of great importance.

*Helicobacter pylori* infection elicits an inﬂammatory cell response, and the severity of mucosal injury appears to be directly correlated with the extent of neutrophil infiltration (Craig et al. 1992; Davies et al. 1994). It has been suggested that activated leukocytes may be responsible for some of the tissue damage seen in cases of *H. pylori* gastritis (Blaser 1992; Olivieri et al. 1993; Kurose et al. 1994) and that activated phagocytes, especially in the presence of *H. pylori* products, contribute to carcinogenesis (Weitzman & Gordon 1990; Wotherspoon et al. 1993). Since the primary consequence of *H. pylori* infection is gastritis, many investigations have centred on the mechanisms involved in the gastric inﬂammatory response (Wallace 1991; Reymunde et al. 1993; Yoshida et al. 1993). It has previously been shown that exposure of human leukocytes to *H. pylori* antigens generates reactive oxygen species (ROS) *in vitro* (Phull et al. 1995). Moreover, Lignell et al. (1999) obtained experimental results which strongly support the theory that ROS may play a part in the pathogenesis of the *H. pylori*-induced chronic gastritis. Other studies carried out by Sanderson et al. (1997) and Zhang et al. (1997a) suggested that antioxidants might play a very important role in protecting the gastric mucosa against *H. pylori*-induced free radicals, their effects on cell and DNA damage and later development of gastric carcinomas. Thus, the use of antioxidants to combat *H. pylori* infection is an attractive new treatment strategy.

*Bryophyllum pinnatum* (Lam.) Kurz (Crassulaceae) is a perennial herb growing widely and used in folkloric medicine in tropical Africa, tropical America, India, China and Australia. The divine herb contains a wide range of active compounds, including alkaloids, phenols, triterpenes, glycosides, flavonoids, steroids, bufadienolides, lipids and organic acids. This plant is widely used in traditional medicine for the treatment of a variety of ailments and it is well known for its hemostatic and wound healing properties. The leaves have been reported to possess a variety of medicinal properties including, antimicrobial, antifungal, antiulcer, antihypertensive, tocolytic, antidiabetic, anti-inflammatory and analgesic (Raymond et al. 2010). Other reported properties include antitumor, sedative and muscle relaxant effects (Raymond et al. 2010). It may also be effective in the treatment of leishmaniasis (Raymond et al. 2010). Despite its pharmacological properties as antiulcerous and gastroprotective, the activity of this plant has not been investigated against *H. pylori,* a major cause of gastric ulcer (Marshall & Windsor 2005). This study was therefore carried out to evaluate the antimicrobial activity of crude methanol and ethyl acetate extracts of the leaves of *Bryophyllum pinnatum* against 6 clinical strains of *Helicobacter pylori in vitro*, and *in vivo* on mouse model. The antioxidant properties of this plant were also investigated in order to explore the possible mechanisms that underlie the observed anti-*H. pylori* effect of this plant.

## Materials and methods

### Bacterial strains

A total of six strains of *H. pylori* were freshly isolated from gastric biopsies of patients with gastric-related morbidities undergoing endoscopy at Laquintinie Hospital in Douala-Cameroon. The study was approved by local ethical committee of Laquintinie Hospital (Approval N^0^ 425/AR/MINSANTE/HLD/SCM/CR) and the specimens were only collected from the patients who had given consent. The isolates were identified by Gram staining and enzymatic activity (catalase, oxidase, urease) (Miendje Deyi et al. 2011). Pure cultures were suspended in Eppendorf tubes containing 1 mL of Brian Heart Infusion (BHI) broth supplemented with 5% horse serum and 20% glycerol and stored at −80 °C until used.

Our previous study on the screening of these clinical strains of *H. pylori* against a variety of antibiotics; β-lactams, aminosides, glycopeptides, phenicoles, tetracycline, macrolides, polymycines, fluoroquinolones, nitroimidazoles and lincosamides, showed that all six isolates were resistant to 58% (11/19) to the antibiotics tested, indicating a multidrug resistance character of the tested strains (Kouitcheu Mabeku et al. 2016). When we consider the regimen (amoxicillin, tetracycline, clarithromycin, metronidazole or ciprofloxacine) most commonly used in the treatment of symptomatic individuals, we noticed that 100 and 67% of the isolates were resistant to amoxicillin and metronidazole, respectively. However, all the strains were susceptible to tetracycline (doxycycline) and ciprofloxacine, thus these antibiotics were used as positive controls in the following tests.

### Animals

Swiss mice weighing 17–23 g obtained from Animal House, University of Dschang-Cameroon were used for the study. The animals were fed with standard pellet diet and water *ad libitum* throughout the experiment. The experimental animals were maintained in a controlled environment (12:12 h light and dark cycle) and temperature (24 °C ± 2 °C). Prior authorization for the use of laboratory animals was obtained from the Cameroon National Ethics Committee (Reg. No. FWA-IRB00001954).

## Field sampling

The leaves of *Bryophyllum pinnatum* were collected in January 2015 in Dschang, Cameroon. The identification of the plant was made by M. Victor Nana of the National Herbarium (Yaoundé, Cameroon) where the voucher specimen was available under the reference number N° 33394 HNC (HNC: Cameroon National Herbarium). Upon collection, the leaves were air dried at room temperature. The dried field sample was ground into a fine powder.

### Preparation of plant-extract

This was carried out by soaking the dried powdered plant (500 g) in a bottle with 7 L of ethyl acetate (EA) or methanol (MeOH) and kept for 72 h. The EA or MeOH mixture was then filtered. The filtrate was concentrated by evaporating ethyl acetate (77 °C) or methanol (65 °C) under reduced pressure using a rotary evaporator (Buchi n° 253271, Swizeland). The extract was further concentrated by allowing it to stand overnight in an oven at 30 °C. The yields of the MeOH and EA extracts were calculated by dividing the weight of the extract obtained by the weight of the dried material plant extracted.

### *In vitro* anti-helicobacter activity of *Bryophyllum pinnatum* extract: determination of MIC and MBC values

Plant extracts were further used for the determination of MICs by INT (*p*-iodonitrotetrazolium chloride) broth microdilution method (Mativandlela et al. 2006). The micro-dilution test was performed in 96 well plates. Two-fold dilutions of the extract were prepared in the test wells in BHI broth supplemented with 5% horse serum (BHI-serum). The final extract concentrations ranged from 2 to 1024 μg/mL. Inoculums (100 μL) at McFarlands turbidity standard 3 from a 48 h colony in Columbia agar supplemented with 5% (v/v) lacked horse blood and 1% (v/v) Vitox was then added to 100 μL of extract-containing culture medium. Control wells were prepared with culture medium and bacterial suspension and broth only, respectively. Ciprofloxacine, doxycycline and metronidazole at concentration ranges of 2–128 μg/mL were used as positive control. The plates were covered with a sterile plate sealer and then agitated with a shaker to mix the contents of the wells and incubated for 3 days at 37 °C under microaerophillic conditions. After incubation, 40 μL of 0.2 mg/mL INT was added to each well, and incubated at 37 °C for an additional period of 30 min. Viable bacteria reduced the yellow dye (INT colour) to pink. MIC was defined as the sample concentration that prevented the colour change of the medium and exhibited complete inhibition of microbial growth. The MBC was determined by adding 50 μL aliquots of the preparations which did not show any growth after incubation during MIC assays, to 150 μL of adequate broth. These preparations were incubated at 37 °C for 72 h under microaerophillic conditions. The MBC was regarded as the lowest concentration of extract, which did not produce a colour change after addition of INT as mentioned above. All the experiments were carried out in triplicate.

### Phytochemical analysis

#### Preliminary phytochemical activity

This was done according to the method proposed by Parekh and Chanda (2008) to determine the presence of alkaloids (Mayer’s, Wagner, Dragendorff’s tests), flavonoids (alkaline reagent, Shinoda), phenolics (lead acetate, alkaline reagent test), triterpenes and steroids (Liberman-Burchard test), saponins (foam test), tannins (gelatine), anthraquinones (ether-chloroform 1:1 v/v, NaOH 10% w/v) and anthocyanins (1% HCl, heating). The results were qualitatively expressed as positive (+) or negative (−).

#### Quantitative phytochemical analysis

##### Estimation of total phenolic content

The total phenolic content of the extract sample was evaluated using an adapted Folin-Ciocalteu colorimetric method (Gonçalves et al. 2013). 0.5 mL of the sample (1 mg/mL) was mixed with 2.5 mL of 0.2 N Folin–Ciocalteau reagent for 5 min and 2.0 mL of 75 g/L sodium carbonate were then added. The absorbance of the reaction was measured at 760 nm with a spectrophotometer (Thermo Scientific BioMates^TM^ 3S), after 2 h of incubation at room temperature. A standard curve was calculated using gallic acid concentrations ranging from 25 to 500 μg/mL in methanol/water (1:1, v/v) and the results were expressed as mg/g gallic acid equivalents (GAE) of dried weight. All the experiments were carried out in triplicate and the results were expressed as mean ± standard deviation (SD).

##### Estimation of total flavonoid content

The total flavonoid content of the extract sample was evaluated using a colorimetric method (Suhartono et al. 2012). Briefly, 0.5 mL of the sample (1 mg/mL) was separately mixed with 1.5 mL of methanol, 0.1 mL of 10% aluminum chloride, 0.1 mL of 1 M potassium acetate, and 2.8 mL of distilled water, and left at room temperature for 30 min. The absorbance of the reaction mixture was measured at 415 nm with a spectrophotometer (Thermo Scientific BioMates^TM^ 3S). A standard curve was prepared using 25 to 500 μg/mL solutions of rutin in methanol. All determinations were carried out in triplicate. Total flavonoid content was expressed in terms of rutin equivalent (RE) mg/g of dry mass sample.

### Antioxidant assay

#### DPPH radical scavenging assay

The free radical-scavenging capacity of the sample was determined using DPPH (Cieśla et al. 2012).Various concentrations of plant-extract (1.62 to 200 μg/mL) were added to 1 mL of the 0.004% methanol solution of DPPH, and the mixture was vortexed vigorously. The tubes were then incubated at room temperature for 30 min in dark, and the absorbance was taken at 517 nm. Vitamin C was taken as known free-radical scavengers. All tests were performed in triplicate, then the percentage inhibition activity was calculated using the following formula, % Inhibition = [1 − (*A*_1_)/*A*_0_] × 100, where *A*_0_ was the absorbance of the control (without extract) and *A*_1_ was the absorbance of the extract or standard. Inhibitory concentration (IC_50_), which was defined as the concentration (μg/mL) of sample required to inhibit the formation of DPPH radicals by 50%, was determined.

#### Determination of reducing power

The reducing power ability of the extract was determined using the method of Adesegun et al. (2008) by mixing 2.5 mL of extract solution of various concentrations; 1.62 to 200 μg/mL with 2.5 mL of phosphate buffer (0.2 M, pH 6.6), 2.5 mL of potassium ferricyanide (1%) and was incubated at 50 °C for 30 min. Trichloroacetic acid (2.5 mL, 10%) was added and centrifuged for 10 min at 2000 rpm and the supernatant (2.5 mL) was then mixed with an equal volumes of distilled water and ferric chloride (0.5 mL, 0.1%).The absorbance was measured at 700 nm against a blank reagent. Vitamin C was used as reference materials. All the tests were performed in triplicate. A higher absorbance indicated a higher reducing power. EC_50_ (effective concentration) values (μg/mL) were calculated and indicate the effective concentration at which the absorbance was 0.5 for reducing power.

#### Hydroxyl radical scavenging assay

The scavenging activity for hydroxyl radicals was measured with Fenton reaction as reported by Yu et al. (2004). Reaction mixture contained 60 μL of 1.0 mM FeCl_2_, 90 μL of 1 mM 1,10-phenanthroline, 2.4 mL of 0.2 M phosphate buffer (pH 7.8), 150 μL of 0.17 M H_2_O_2_, and 1.0 mL of extract at various concentrations (1.62 to 200 μg/mL). The reaction started when H_2_O_2_ was added. After incubation at room temperature for 5 min, the absorbance of the mixture at 560 nm was measured with spectrophotometer (Thermo Scientific BioMates^TM^ 3S) which vitamin C (1.62 to 200 μg/mL) was used as a standard. All tests were performed in triplicate. The percentage inhibition of hydroxyl scavenging activity was calculated using the following formula; % Inhibition = [1 − (*A*_1_)/*A*_0_] × 100, where *A*_0_ was the absorbance of the control (without extract) and *A*_1_ was the absorbance of the extract or standard.

### *In vivo* assessment of anti-*H. pylori* activity

#### Inoculation of experimental animals

Sixty mice were inoculated orally through a feeding tube four times at 2-day intervals with 0.2 mL of an *H. pylori* α2 suspension containing 10^8^ CFU/mL after an acclimatization period of seven day. Each inoculation was preceded by a 12 h fast. The day of inoculation was defined as day 0 and subsequent days as day 1, day 2, and day 3 up to day 21. Twelve animals initially uninfected were treated with vehicle (200 μL of a sterile solution of 0.25% Tween 80) and consider as a normal control group.

#### Distribution of animals

The *H. pylori*-inoculated mice were divided into five groups of 12 animals and allowed to rest for 24 h after the last inoculation.

Negative control (Group 1): In this group, animals were infected with *H. pylori* and then treated with vehicle (200 μL of a sterile solution of 0.25% Tween 80).

Tested group (Group 2): animals were infected with *H. pylori* and then treated with *Bryophyllum pinnatum* extract, 125 mg/kg for 7 days administered orally.

Tested group (Group 3): animals were infected with *H. pylori* and then treated with *Bryophyllum pinnatum* extract, 250 mg/kg for 7 days administered orally.

Tested group (Group 4): animals were infected with *H. pylori* and then treated with *Bryophyllum pinnatum* extract, 500 mg/kg for 7 days administered orally.

Positive control (Group 5) this group, initially infected with *H. pylori* and then treated with standard drug ciprofloxacine, 500 mg/kg for 7 days administered orally.

Half of the animals from each group were sacrificed 1 day after the cessation of treatment, and the other half were sacrificed 07 days after the cessation of treatment. Mice were killed with ethyl ether, and their stomachs were collected. The stomachs were opened through the longer curvature using sterile surgical instruments and used for culturing *H. pylori*.

#### Culture

Stomach biopsies covering all subtypes of mucosa were scraped off and homogenized with 0.5 mL of PBS in a 1.5 mL Eppendorf tube. The homogenized sample was serially diluted 10-fold. Each 0.1 mL of homogenate was plated on Columbia agar supplemented with 5% (v/v) lacked horse blood and 1% (v/v) Vitox, and incubated at 37 °C for 2 to 3 days under microaerophilic conditions (Miendje Deyi et al. 2011). The presence of *H. pylori* on the culture plates was confirmed by urease, catalase, and oxidase testing, and Gram staining. The *H. pylori* colonies were counted and expressed as log_10_ CFU per mL of homogenate.

### Statistical analysis

The results were expressed as means ± standard deviation (SD). Bacterial densities were expressed as log_10_ values before analysis. Data were statistically evaluated using the analysis of variance following by the paired Student’s *t-*test. The differences between groups were considered significant at *p* < 0.05.

## Results

### MIC and MBC determination of plant-extracts and antibiotics

As it is in the susceptibility test using disc diffusion method, the strains were susceptible to ciprofloxacin and doxycicline with the MIC value ranged of 2 to 64 μg/mL, but resistant to metronidazole. Methanol extract of *B. pinnatum* was the potent crude extract (MIC/MBC = 32/256 μg/mL) against *H. pylori α2*. This activity was similar to that obtained with ciprofloxacin and doxycycline against the same clinical strains. Only one of the six isolates tested, *H. pylori α2* was susceptible to crude extracts tested (Table 1). This is the reason why this extract was used in further assessment.

**Table 1.** MIC/MBC of ethyl acetate and methanol crude extracts of *B. Pinnatum* and antibiotics (μg/mL).

Clinical strains	Crude extracts/Antibiotics				
	*Bryophyllum pinnatum*(MeOH*)*	*Bryophyllum pinnatum* (AE)	Ciprofloxacin	Doxycycline	Metronidazole
*Helicobacter pylori α1*	128/128	256/256	8/8	64/64	>128
*Helicobacter pylori α2*	32/256	256/256	32/32	32/32	>128
*Helicobacter pylori α3*	512/1024	>1024	2/2	32/32	>128
*Helicobacter pylori α4*	>1024	>1024	<2	32/32	>128
*Helicobacter pylori α5*	128/512	>1024	16/16	4/16	>128
*Helicobacter pylori α6*	>1024	>1024	8/8	32/32	>128

All the values given in the table are means of triplicates determinations. EA: ethyl acetate; MeOH: methanol.

### Phytochemical screening

The yields of extraction were 4.67% and 6.32% w/w for methanol and ethyl acetate extracts, respectively. The results of the preliminary phytochemical screening of methanol extract of *B. pinnatum* revealed the presence of alkaloids, phenols, flavonoids, tannins, anthraquinones and steroids (Table 2). The total flavonoids and phenol content present in this plant-extract were 93.23 ± 8.23 RE μg/g and 406 ± 11.2 GAE μg/g, respectively.

**Table 2.** Phytochemical screening of methanol extract of *B. pinnatum*.

Compounds	Methanol extract of*B. pinnatum*
Alkaloids	+
Flavonoids	+
Phenolics	+
Saponins	–
Tannins	+
Triterpenes	–
Anthraquinones	+
Anthocyanines	–
Steroids	+

The plus sign (+) indicates that there is the presence of a given compound while the minus (−) shows a given compound is missing.

### Antioxidant assay

#### Scavenging effect on 2, 2-diphenyl-1-picryl hydrazyl radical

DPPH radical scavenging activity increased with an increase in extract concentration. This activity was slightly comparable to that of Vitamin C as standard. The IC_50_ values of the tested plant-extract and Vitamin C were 25.31 ± 0.34 and 6.08 ± 0.40 μg/mL, respectively (Table 3).

**Table 3.** Antioxidant activity of *Bryophyllum pinnatum* methanol extract and vitamin C.

Plant extract and standard	DPPH (IC_50_ μg/mL ± SD)	Reducing power (EC_50_ μg/mL ± SD)	Hydroxyl radical (IC_50_ μg/mL ± SD)
*Bryophyllum pinnatum*	25.31 ± 0.34^ab^	55.94 ± 0.68^b^	11.18 ± 0.74
Vitamin C	6.08 ± 0.40^a^	15.32 ± 0.11^a^	9.71 ± 0.40

Data are means ± standard deviation (*n* = 3 tests). The values in the same column with different superscript letter (^a^ or ^b^) are significantly different (*p* < 0.05).

#### Reducing power activity

It was found that the sample was significantly less active than the standard as far as hydroxyl radical scavenging activity is concerned. The IC_50_ value was found to be 55.94 ± 0.68 μg/mL for sample while that of vitamin C was 15.32 ± 0.11 μg/mL (Table 3).

#### Hydroxyl radical scavenging activity

The reducing potential of the sample was compared to that of vitamin C. The EC_50_ value of reducing power for sample and vitamin C were 11.18 ± 0.74 and 9.71 ± 0.40 μg/mL, respectively (Table 3).

### *In vivo* anti-helicobacter assessment

#### Culture

After stomach biopsies culture, the presence of *H. pylori* on the plates was confirmed by urease, catalase, oxidase testing and Gram staining. The result obtained from animal of each group was expressed as a percentage of negative tests and compared to the control infected group. All non-inoculated mice (normal group) were *H. pylori* negative in culture. Both *B. pinnatum* (dose from 125 to 500 mg/kg) and ciprofloxacin significantly reduced the presence of *H. pylori* organisms in gastric tissue on day one and seven after the cessation of treatment as compared to what was observed with the control-infected mice treated with vehicle (Table 4).

**Table 4.** Effect of different dose of *B. pinnatum* and ciprofloxacin on the percentage (%) of *H. pylori* tested negative animals.

Treatment group (mg/kg)	Negative test (%)							
	1 day after cessation of treatment				7 days after cessation of treatment			
	Urease	Catalase	Oxidase	Gram staining	Urease	Catalase	Oxidase	Gram staining
Normal	100	100	100	100	100	100	100	100
*Helicobacter pylori* + vehicle	17	0.00	0.00	17	0.00	0.00	0.00	0.00
*Helicobacter pylori* + *B. pin*, 125	67	67	83	50	67	100	100	67
*Helicobacter pylori* + *B. pin,* 250	67	83	83	50	83	100	100	67
*Helicobacter pylori* + *B. pin*, 500	83	83	100	67	83	100	100	83
*Helicobacter pylori* + Ciprof, 500	83	100	100	83	100	100	100	100

All the percentages given in the table are means of the six animals (*n* = 6) that tested negative per group. *B. pin: Bryophyllum pinnatum*; Ciprof: ciprofloxacin.

#### Bacterial load

**Figure 1.**
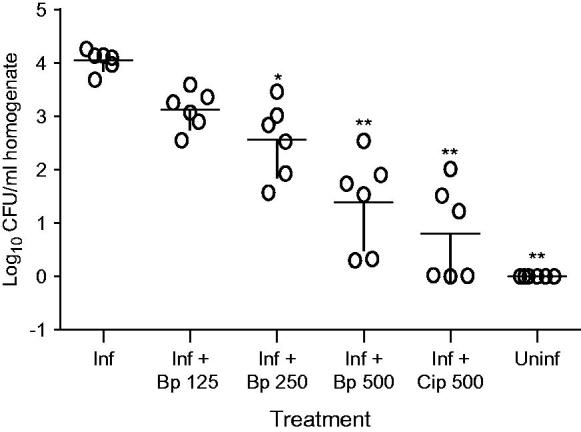
Effects of different treatment on the recovery of *H. pylori* from infected mice. Each circle represents the bacterial count for one animal. (Inf): infected + vehicle; (Inf + Bp 125): infected + *B pinnatum* 125 mg/kg; (Inf + Bp 250): infected + *B pinnatum* 250 mg/kg; (Inf + Bp 500): infected + *B pinnatum* 500 mg/kg; (Inf + Cp 500): infected + ciprofloxacin 500 mg/kg; (Uninf): normal group. Data column of the different treatment with superscript * are significantly different compared with infected control group (*p < 0.05; **p < 0.01).

Inoculation of mice with *H. pylori* resulted in stable infections of the gastric mucosa as shown by the number of bacterial count. Treatment of the infected mice with *B. pinnatum* extract and ciprofloxacin significantly reduced bacterial load of gastric mucosa in infected mice as compared to untreated, infected mice (mean CFU 85.91 ± 52.91 for *B. pinnatum* 500 mg/kg and 25.74 ± 16.15 for ciprofloxacin 500 mg/kg versus 11883 ± 1831 for untreated infected mice) (Figure 1).

## Discussion

In this study, the antibacterial activity of methanol extract of *B. pinnatum* was evaluated on *H. pylori* infected mice. One interest of our study was also to evaluate the possible mechanisms that underlie the observed anti-*H. pylori* effect of the tested plant extract.

*In vitro*, the anti-*Helicobacter* activity of the methanol and ethyl acetate extract of *B. pinnatum* showed MIC values that ranged from 32 to >1024 μg/mL and 256 to >1024 μg/mL, respectively. The activity of the methanol extract demonstrated herein indicates that the antimicrobial potential components of the tested plant are more soluble in methanol. The ability of the methanol to extract antimicrobial potential compounds reported in this study is in line with many others previous studies reporting that methanol is a better solvent for extraction of antimicrobial substances from medicinal plants than water, ethanol, ethyl acetate or hexane (Ezekiel et al. 2009; Kouitcheu Mabeku et al. 2011). Phytochemicals are routinely classified as antimicrobials on the basis of susceptibility tests that produce MIC in the range of 100 to 1000 μg/mL (Simões et al. 2009). Activity is considered to be significant if MIC values are below 100 μg/mL for crude extract and moderate when 100 < MIC <625 μg/mL (Kuete 2010). Taking this into account, the activity recorded with *B. pinnatum* methanol extract on *H. pylori* can be considered significant. MIC value for the methanol extract of *B. pinnatum* against *H. pylori* α2 was 32 μg/mL (Table 1) and was compared with those of ciprofloxacin and doxycycline in contrast to that of metronidazole. *Helicobacter pylori* resistance to metronidazole is at alarming proportions in many developing countries, in which resistance up to 100% have been reported (Aboderin et al. 2007; Tanih et al. 2010). It should therefore not be surprising that the activity of the *B. pinnatum* extract in this study was better than metronidazole. This is quite remarkable particularly as standard antibiotics are in the purified and concentrated form whereas the extracts are crude and may harbour both pharmacologically and non-pharmacologically active compounds with the chance of some compounds having a masking effect over others. This observation highlighting the importance of the results reported herein. The antimicrobial activities of 5-methyl-4,5,7-trihydroxyl flavones and 4,3,5,7-tetrahydroxy-5-methyl-5-propenamine anthocyanidines, two novel flavonoids isolated from this plant against *Pseudomonas aeruginosa, Klebsiella pneumonia, Escherichia coli, Staphylococcus aureus, Candida albicans* and *Aspergillus niger*, have been reported (Okwu & Nnamdi 2011) and the present study provides additional data on the ability of this plant to fight *Helicobacter pylori.* Phytochemical screening of methanol extract of *B. pinnatum* revealed the presence of alkaloids, phenols, flavonoids, tannins, anthraquinones, anthocyanins and steroids (Table 2). The result of the phytochemical screening obtained herein correlates with those of some previous studies showing that *B. pinnatum* is rich in alkaloids, triterpenes, glycosides, flavonoids, cardienolides, steroids, bufadienolides and lipids (Costa et al. 1995). The presence of these chemical compounds in the tested extract may partially explain its anti-*Helicobacter* effect, since it has clearly been demonstrated that several compounds belonging to the investigated classes of metabolites showed antibacterial activities (Bruneton 1999; Cowan 1999; Kouitcheu Mabeku et al. 2011).

*In vivo*, methanol extract of *B. pinnatum* showed a concentration dependent effect against *H. pylori* (Table 4 and Figure 1). The culture of gastric biopsies of infected mice showed a lower percentage of negativity to the confirmation tests for the presence of *H. pylori* used (urease, oxidase and catalase tests and Gram staining). Treatment of infected animal with plant-extract significantly increases the percentage of negativity from 67 to 83%, respectively, at 125 and 500 mg/kg doses, as concern urease test (Table 4). This inhibiting activity against *H. pylori* was also confirmed by the *H pylori* count in gastric biopsies culture, where the decrease in bacterial population was observed with both extract (85.91 ± 52.91 CFU) and standard antibiotic (25.74 ± 16.15 CFU) used (Figure 1). The anti-*Helicobacter* effect of the plant-extract obtained *in vitro* was also effective *in vivo*, indicating the acid stability of antimicrobial components of this extract, since poor acid stability render many compounds endowed with *in vitro* anti-*Helicobacter* properties ineffective *in vivo* (Haas et al. 1990; Skirrow 1992; Krakowka et al. 1998). The activity of the tested plant-extract demonstrated herein is very important when considering the medicinal importance of the tested bacteria. In fact, *H. pylori* is the principal cause of type B gastritis, peptic ulcer disease, gastric adenocarcinoma and MALT-lymphoma and has been classified as a Class I carcinogen by the World Health Organization (Manyi-Loh et al. 2010).

*Helicobacter pylori* colonizes the gastric antrum and sites of gastric metaplasia in the duodenum, and induces local inﬂammation with activated neutrophils (Carrick et al. 1989; Blaser 1990; Lamarque et al. 1998). The processes by which *H. pylori* infection may provoke damage in the stomach and duodenum are unclear, but the bacteria could release soluble cytotoxic factors that activate inﬂammatory cells such as neutrophils, that are implicated in tissue damage (Nielsen & Andersen 1992) and microvascular leakage (Kurose et al. 1994). Such activation of neutrophils could provoke the release of free radicals and other reactive species, including superoxide (Mooney et al. 1991) and nitric oxide (NO) (McCall et al. 1989). *Helicobacter pylori*-infected individuals show high-oxidative stress and high levels of ROMs in their gastric mucosae and an increased gastric antioxidative capacity after the eradication of *H. pylori* (Khaled & Sarker 1998). Jarosz et al. (1998) reported that a high-daily dose of vitamin C as standard antioxidant for 4 weeks (5 g per day) given to *H. pylori*-infected patients with chronic gastritis resulted in apparent *H. pylori* eradication in 30% of treated patients. The total antioxidant activity of the extracts is constituted by individual activities of each of the antioxidant compounds. Moreover, these compounds render their effects via different mechanisms such as radical scavenging, metal chelating activity, inhibition of lipid peroxidation, quenching of singleton oxygen, etc., to act as antioxidant. In the present paper, we have evaluated the antioxidant activity and free radical scavenging activity of the methanol extract of *B. pinnatum* through DPPH radical scavenging activity, hydroxyl radical scavenging activity and reducing power assay.

DPPH assay estimates the ability of sample to scavenge free radicals species capable of causing damage to natural macromolecules, such as nucleic acids, polysaccharides and lipids (Issa et al. 2006). We noticed that the antiradical activity of the methanol extract of *B pinnatum* was found to be slightly comparable to that of vitamin C as far as the DPPH assay was concerned (Table 3).

The reducing properties are generally associated with the presence of reductants (Meir et al. 1995) which have been shown to exert antioxidant action by breaking the free radical chain by donating a hydrogen atom (Schimada et al. 1992). In this study, the tested plant-extract was significantly less active than the standard used (Table 3).

Hydroxyl radical is highly reactive oxygen radical formed from the reaction of various hydroperoxides with transition metal ions. It attacks proteins, DNA, polyunsaturated fatty acid in membranes, and most biological molecules it contacts (Nabavi 2009) and is known to be capable of abstracting hydrogen atoms from membrane lipids and bringing about peroxidic reaction of the lipids. We noticed that, the reducing potential of the plant-extract was similar to that of vitamin C (Table 3).

Our data suggest that the methanol extract of *B. pinnatum* possess antioxidant activities. Flavonoid and phenolic content of this plant-extract was found to be 93.23 ± 8.23 (RE) and 406 ± 11.2 (GE) μg/g dry weight. This result shows an outstanding antioxidant activity of *B. pinnatum* since phenolics and flavonoids, in general, constitute a major group of compounds, which act as primary antioxidants, are known to react with hydroxyl radicals, superoxide anion radicals and lipid peroxyradicals (Velioglu et al. 1998; Adesegun et al. 2009; Anwar et al. 2010; Bouba et al. 2010). Moreover, syringic acid, caffeic acid, 4-hydroxy-3-methoxy-cinnamic acid, 4-hydroxybenzoic acid, *p*-hydroxycinnamic acid, *p*-coumaric acid, ferulic acid, protocatechuic acid, phosphoenolpyruvate, protocatechuic acid are phenols, phenylpropanoids and flavonoids compounds isolated from aerial parts of this plant (Costa et al. 1995).

## Conclusion

In addition to its anti-*Helicobacter pylori* activity, *B. pinnatum* methanol extract acts as an antioxidant that protects against gastric mucosa damage induced by ROMs generated via *H. pylori* infection. The activity of the methanol extract demonstrated herein indicates that the active components of *B. pinnatum* are more soluble in methanol and have good anti-*H. pylori* and antioxidant potential, which if investigated further may lead to the isolation of potentially useful compounds for the treatment of *H. pylori* infections.
